# Effects of a staff-led multicomponent physical activity intervention on preschooler's fundamental motor skills and physical fitness: The ACTNOW cluster-randomized controlled trial

**DOI:** 10.1186/s12966-024-01616-4

**Published:** 2024-07-03

**Authors:** Elisabeth Straume Haugland, Ada Kristine Ofrim Nilsen, Kristoffer Buene Vabø, Caterina Pesce, John Bartholomew, Anthony David Okely, Hege Eikeland Tjomsland, Katrine Nyvoll Aadland, Eivind Aadland

**Affiliations:** 1 Faculty of Education, Arts and Sports, Department of Sport, Food and Natural Sciences, Western Norway University of Applied Sciences, Sogndal, Norway; 2 Department of Movement, Human and Health Sciences, University of Rome “Foro Italico”, Rome, Italy; 3 Department of Kinesiology and Health Education, The University of Texas at Austin, Austin, TX USA; 4 Early Start and School of Education, University of Wollongong, Wollongong, NSW Australia

**Keywords:** Preschool, Professional development, Physical activity, Motor skills, Physical fitness

## Abstract

**Background:**

Fundamental motor skills (FMS) and physical fitness (FIT) play important roles in child development and provide a foundation for lifelong participation in physical activity (PA). Unfortunately, many children have suboptimal levels of PA, FMS, and FIT. The Active Learning Norwegian Preschool(er)s (ACTNOW) study investigated the effects of a staff-led PA intervention on FMS, FIT, and PA in 3–5-year-old children.

**Methods:**

Preschools in Western Norway having ≥ six 3–4-year-old children were invited (*n* = 56). Of these, 46 agreed to participate and were cluster-randomized into an intervention (*n* = 23 preschools [381 children, 3.8 yrs., 55% boys]) or a control group (*n* = 23 [438, 3.7 yrs., 52% boys]). Intervention preschools participated in an 18-month PA intervention involving a 7-month staff professional development between 2019 and 2022, amounting to 50 h, including face-to-face seminars, webinars, and digital lectures. Primary outcomes in ACTNOW were cognition variables, whereas this study investigated effects on secondary outcomes. FMS was measured through 9 items covering locomotor, object control, and balance skills. FIT was assessed as motor fitness (4 × 10 shuttle-run test) and upper and lower muscular strength (handgrip and standing long jump). PA was measured with accelerometers (ActiGraph GT3X +). All measures took place at baseline, 7-, and 18-month follow-up. Effects were analysed using a repeated measures linear mixed model with child and preschool as random effects and with adjustment for baseline scores.

**Results:**

Participants in the intervention preschools showed positive, significant effects for object control skills at 7 months (standardized effect size (ES) = 0.17) and locomotor skills at 18 months (ES = 0.21) relative to controls. A negative effect was found for handgrip strength (ES = -0.16) at 7 months. No effects were found for balance skills, standing long jump, or motor fitness. During preschool hours, sedentary time decreased (ES = -0.18), and light (ES = 0.14) and moderate-to-vigorous PA (ES = 0.16) increased at 7 months, whereas light PA decreased at 18 months (ES = -0.15), for intervention vs control. No effects were found for other intensities or full day PA.

**Conclusions:**

The ACTNOW intervention improved some FMS outcomes and increased PA short-term. Further research is needed to investigate how to improve effectiveness of staff-led PA interventions and achieve sustainable improvements in children’s PA, FMS, and FIT.

**Trial registration:**

Clinicaltrials.gov, identifier NCT04048967, registered August 7, 2019.

**Funding:**

ACTNOW was supported by the Research Council of Norway (grant number 287903), the County Governor of Sogn og Fjordane, the Sparebanken Sogn og Fjordane Foundation, and the Western Norway University of Applied Sciences.

**Supplementary Information:**

The online version contains supplementary material available at 10.1186/s12966-024-01616-4.

## Introduction

The early years are critical for the establishment of healthy habits, including sufficient levels of physical activity (PA) [1], which is understood as bodily movement produced by the skeletal muscles that results in energy expenditure above resting levels [2, 3]. As PA is favorably associated with fundamental motor skills (FMS) and physical fitness (FIT) in childhood [1, 4, 5], and FMS and FIT are considered to be “building blocks” for more advanced skills [6] and prerequisites of PA participation [7], it is important to provide varied movement opportunities for children as early as preschool age [8, 9]. FMS are often categorized by the domains locomotor, object control, and balance skills [6, 10], while FIT includes components of muscular strength, motor fitness, and cardiovascular fitness [7]. According to the World Health Organization (WHO) and Norwegian guidelines, 3–5 year old children should accumulate a minimum of 180 min of daily PA, of which 60 min should be in moderate-to-vigorous-intensity (MVPA) [3, 11]. A recent meta-analysis showed that 60% of preschool children from multiple countries adhere to the overall PA recommendation. Yet, the study underlines the significant variability in PA levels for different accelerometer cut points, ranging from 4–100% achievement for the overall PA guideline [12]. Unfortunately, many children demonstrate sub-optimal levels of PA [12–15], FMS [16, 17], and FIT [18, 19], which is a concern given that movement behaviors are likely to track into adolescence and adulthood [20–22]. Hence, interventions to promote optimal PA behavior should be initiated in the early years [23] and include efforts to improve FMS and FIT. As 97% of children in Norway aged 3–5 years attend preschool full-time [24], it is a critical setting for influencing child behavior, including PA participation that may impact development. Developing potentially effective and scalable interventions to improve FMS and FIT in children through high-quality PA in preschool is an important public health approach [25].

There is evidence for the benefits of PA on some aspects of FMS and FIT. Systematic reviews and meta-analyses have shown moderate to large effects of PA interventions on locomotor and object control skills in preschoolers [9, 26–28]. In contrast, there is limited evidence regarding the effect of PA on balance skills [29], although they are recognized as an equally important component of FMS [29]. Regarding FIT, a meta-analysis showed a small effect on preschoolers’ lower-body muscular strength and motor fitness following PA interventions [30], also confirmed in more recent RCTs [31–33], whereas findings are inconsistent for upper-body muscle strength [31–34]. Thus, broad impact of PA interventions should be investigated across various FMS and FIT measures.

To have a population-level impact, PA interventions in children must be pragmatic, effectively implemented and sustained under real-world conditions [35]. Of those intervention studies that show positive effects in preschoolers, many are of low quality with only short-term follow-ups [9, 27, 30]. Furthermore, effective interventions are characterized by having experts delivering the interventions, whereas staff-led interventions have shown poor effectiveness [9, 26, 32, 36]. Li et al. [27] showed better effects of interventions with trained preschool teachers rather than ordinary preschool teachers (i.e., teachers who have not received any specific PA training). However, the amount of staff training provided in previous studies is often insufficient to enhance staff competence which results in any benefits of an intervention being small and not sustainable [27, 28, 30]. In Norway, less than 50% of preschool staff are certified teachers [24], underlining the importance of enhancing staff competence in PA promotion through intensive training [37]. Furthermore, tailoring interventions to each preschool’s contextual factors have been deemed essential for long-term intervention effectiveness and sustainability [28, 37, 38].

Given the emerging relevance of having trained preschool teachers [36] and considering school context-specific factors [38], this study aimed to investigate 7- and 18-month effects of a staff PA professional development intervention on FMS, FIT, and PA in preschool children. Secondary aims were to investigate moderation by age, sex, and baseline performance.

## Methods and materials

A description of the ACTNOW protocol has been published [39]. In the following, only procedures relevant to the present analyses will be described.

### Design and participants

The ACTNOW study is a two-armed, 18-month, cluster RCT with random allocation at the preschool level using a 1:1 ratio. The primary outcomes in ACTNOW were children’s cognition variables, while this study investigated the secondary outcomes FMS, FIT, and PA. ACTNOW was designed to detect statistically significant standardized effect sizes (ESs) (Cohen’s d) between 0.20 and 0.30 [39]. A third party was responsible for randomization using a random number generator (Stata/SE 15.1, StataCorp LLC, College Station, TX, United States). Block randomization with block sizes of four and six was used. Fifty-six preschools from Western Norway having ≥ six 3–4-year-old children were invited to participate. All children born between 2014–2017 (i.e., aged 3–5 years at baseline) within participating preschools were invited to participate. The recruitment and intervention were conducted in two waves of different preschool cohorts, the first in 2019–2021 and the second in 2020–2022. Data collection was performed at baseline (prior to randomization), at 7-months (i.e., at the end of the intensive phase of the professional development), and at 18-month follow-up (i.e., one year after the intensive phase of the professional development was completed). Researchers and assessors were as far as possible blinded for group allocation during the data collection.

Children were recruited through preschools, and an information video, poster, and written information explaining the project’s purpose and measurements were shared with the preschool directors who forwarded the information to parents. Children’s parent(s)/guardian(s) provided written consent for child participation prior to testing. The children were informed at their level of understanding, and all testing was done in familiar environments at their respective preschools. The Norwegian Center for Research Data (NSD, reference number 248220) and the Western Norway University of Applied Sciences institutional ethics committee approved the study. All procedures and methods conform to the ethical guidelines defined by the World Medical Association`s Declaration of Helsinki and its subsequent revisions [40]. The study was registered in clinicaltrials.gov on August 7, 2019 (ID: NCT04048967) (https://clinicaltrials.gov/ct2/show/NCT04048967?term=actnow&rank=1).

### The intervention

The intervention was conducted on two levels: *the preschool level* and *the child level,* placing the preschool as an influential institution for child development according to the socioecological model by McLeroy and colleagues [41]. From each preschool, the director, and a minimum of one teacher from each preschool department participated in a 7-month professional development structured as a 15-credit (i.e., equates to a half-term in course work) education module at master’s degree level (qualifying for credits were optional). In total, 77 directors and teachers participated. The norm for pedagogical staffing in Norwegian preschools requires at least one pedagogical leader per fourteen children over the age of three [24].

The aim was to enhance staff competence on 1) physically active play and its relevance for child development and 2) planning and implementation of the ACTNOW-intervention within the preschool, to increase capacity to intervene at child level [39]. The professional development was approximately 50 h in total, consisting of six face-to-face seminars (four on the university campus and two in each preschool), two webinars (4 h in total), and nine digital lectures (7 lectures of 20 min each, and 2 lectures of 1 h) (see details in Additional file 1). One of the original face-to-face seminars in both waves had to be digital due to regulations during the Covid-19-pandemic. To support staff in delivering the intervention, preschools received tools consisting of portable play equipment of approximately 500 Euro per department, and an online PA toolbox consisting of various activities and resources (https://activeinpreschool.com/). A one-day booster session was held one year after startup. Control preschools upheld their normal practices and received the portable equipment and access to the online toolbox after the study was completed.

At the child level the intervention consisted of four core components delivered by the preschool staff with the aim of promoting whole-child development, which integrated various aspects of physically active play that simultaneously affected physical and mental developmental outcomes: 1) PA of moderate-to-vigorous intensity (60 min/day), 2) motor challenging PA (90 min/week), 3) cognitively engaging PA play (90 min/week), and 4) physically active learning (90 min/week). This was delivered in different ways, from child-initiated or directed free play to structured staff-led activities. A flexible intervention approach allowed researchers and preschool staff to co-create the intervention and staff to integrate the components into preschools´ everyday routine tailored to their specific, contextual factors. As opposed to a rigid “PA program”, a flexible and co-created approach is recommended to facilitate sustainable changes within educational contexts [38, 42].

### Measures

#### Motor skills

FMS was evaluated using a modified test battery [43] guided by the “Test of Gross Motor Development 3” (TGMD-3) [44] and the “Preschooler Gross Motor Quality Scale” (PGMQS) [45]. The test battery combines locomotor- (running, jumping, hopping) and object control (overhand throw, catch, kick) skills from the TGMD-3 and balance skills (single leg standing, walking forwards and backwards on a line) from PGMQS. The structural validity of the test battery is acceptable for this age group, despite commonality between locomotor and object control skills [43]. FMS was tested and scored according to the protocols of the TGMD-3 and PGMQS [44, 45]. One instructor explained and demonstrated each skill, while a separate assessor scored the child performance. Children were scored quantitatively based on the evaluation of whether or not the child demonstrated process criteria using original scoring procedures (“1” or “0” points, respectively) [44, 45]. Children had one test attempt per skill before performing each skill twice in a standardized order, where scores from both trials were summed, scoring 0–2 points per criteria (i.e., sum score between 0 (lower skill level) and 22 (object control skills) or 24 (locomotion and balance skills). Inter-rater reliability (IRR) based on video-scoring of 22 children was 0.76–0.85 across subdomains after adjusting for assessor [43]. Due to a certain variation between raters, calculated estimates based on IRR-scores for each assessor were retracted from the unadjusted scores for each FMS domain. By doing this we accounted for each rater’s individual contribution to the score.

#### Physical fitness

FIT assessment included handgrip strength, standing long jump, and motor fitness, according to the Assessing FITness in PREschoolers (PREFIT) test battery [46]. PREFIT is designed explicitly for preschool populations, and has good reliability in young children [47, 48]. Handgrip strength was measured twice for each hand with a hand dynamometer (TKK 5001 Grip A, analogue model 577, Takey, Tokio), with a standardized grip span of 4.0 cm [49, 50]. We used the highest (kg) of the four scores (two left hand, two right hand) for analyses. Lower body muscle strength was measured by standing long jump, where children were instructed to jump as far as possible from a standing position with a two-footed take-off and landing. The best of two valid performances were used, reported in cm. A 4 × 10 m shuttle run test was used to measure motor fitness, where children were instructed to run as fast as possible back and forth between cones placed 10 m apart. This was performed twice and results were reported in seconds, using the best time for analyses.

#### Physical activity and sedentary time

PA and sedentary time (SED) were assessed objectively using ActiGraph GT3X + accelerometers (ActiGraph, LLC, Pensacola, Florida, USA) [51], which are widely used and validated [52, 53]. Children were asked to wear the accelerometer on their right hip 24 h/day for seven consecutive days (except during water-based activities). The sampling rate was set to 30 Hz, and data were analysed using 1-s epochs [54] using a custom-made script in MATLAB (MathWorks, Massachusetts, USA). Non-wear time was defined as consecutive periods of ≥ 20 min of zero counts [55]. Total PA (hours 06:00–22:00) and PA during preschool hours (hours 08:30–15:30) were analysed to specifically assess intervention effects. For total PA, children had to have ≥ 8 h/day of wear time for ≥ 3 valid weekdays and ≥ 1 valid weekend day to be included in analyses [56]. During preschool hours, children had to have ≥ 5 h/day for ≥ 3 weekdays to be included. PA was reported as total PA (counts per minute [cpm]) and intensity-specific PA and SED as determined using the Evenson et al. [57] thresholds (SED (≤ 100 cpm), light PA (101–2295 cpm), moderate PA (MPA) (2296–4011), vigorous PA (VPA) (≥ 4012 cpm), and MVPA (min/day) (≥ 2296 cpm)).

#### Anthropometry and demography

Parental education level (highest level of mother/father used) was reported by parent(s)/guardian(s) through a questionnaire and used as a proxy for socioeconomic status (SES). Responses were categorized into 1) upper/lower secondary school, 2) university < 4 years and 3) university ≥ 4 years. Children’s weight, height, and waist circumference were assessed according to the PREFIT battery [46]. Children’s weight was measured to the nearest 0.1 kg using a Seca 899-scale (SECA GmbH, Hamburg, Germany). Height was measured to the nearest 0.1 cm using a Seca 217 (SECA GmbH, Hamburg, Germany). BMI (kg m^2^) was calculated. Normal weight, overweight, and obesity were defined according to the cut points by Cole et al. [58]. Waist circumference were measured to the nearest 0.5 cm with a Seca 201 ergonomic circumference measuring tape (SECA GmbH, Hamburg, Germany).

### Adherence to intervention

We assessed adherence to the intervention by reporting the proportion of preschools represented and teachers attending seminars and completing written tasks (e.g., hand-in of project description and monthly PA plans) and by rating their intervention implementation on a 3-point scale (poor, fair, or good) based on experiences of overall commitment and management by researchers responsible for the professional development. Further, after completing the intervention, preschool teachers participating in the professional development reported the level of integration of the intervention model in daily preschool practices on a 5-point scale (from very low to very high integration). We also considered children’s PA levels as a measure of intervention adherence.

### Statistical analyses

Participants’ characteristics, PA, SED, FMS, and FIT were reported as frequencies or means and standard deviations (SD). Outcomes and analyses were determined prior to intervention [39]. Intervention effects were analysed using an intention-to-treat principle [59], meaning that all children with data were included irrespective of them receiving the intervention or not. Analyses were performed using a repeated measures linear mixed model (Eq. 1) including all three timepoints (baseline, 7-month-, and 18-month follow-up) and random intercepts for child and preschool [60].

1 $${Y}_{t} = {\beta }_{0}+{\beta }_{1}{dummytime}_{1}+{\beta }_{2}{dummytime}_{2}+ {\beta }_{3}{dummytime}_{1}*group + {\beta }_{4}{dummytime}_{2}*group$$

Effect estimates were derived from testing the interaction group*time for 7-month (*β*_*3*_*dummytime*_1_*group) and 18-month *(β*_*4*_*dummytime*_2_*group) follow-up. All analyses were adjusted for baseline differences by excluding the main effect of group from the model [60]. PA analyses were additionally adjusted for accelerometer wear time. Effect estimates were reported as regression coefficients (*β*), with 95% confidence intervals (CI), *p*-values, ICCs for the clustering effect of preschool, and standardized ESs. Secondary analyses included investigation of effect moderation by sex, baseline performance (median split), age, and per-protocol analyses. Effect moderations were assessed by running the analysis as described above in subgroups (i.e., sex, age, and baseline performance). Subgroup effects were interpreted as significantly different if 83.4% CIs did not overlap [61, 62]. Standardized ESs were derived by calculating mean differences by pooled SDs for subgroups.

We performed per-protocol analyses for preschools demonstrating acceptable intervention adherence based on three criteria: 1) ≥ 80% participation at the face-to-face seminars and completion of written tasks during the intervention, 2) preschools self-reporting high or very high integration of the intervention model and 3) researchers᾽ ratings of preschools᾽ commitment and management during the intervention being fair or good. We also performed association analyses between changes in children’s PA (adjusted for changes in wear time) and changes in FMS and FIT over 18 months using a mixed model including random intercept for preschool. All analyses were performed using IBM SPSS v. 28 (IBM SPSS Statistics for Windows, Armonk, NY; IBM Corp., USA). Statistical significance was set to *p* $\le$ 0.05.

## Results

### Sample characteristics

**Fig. 1:**
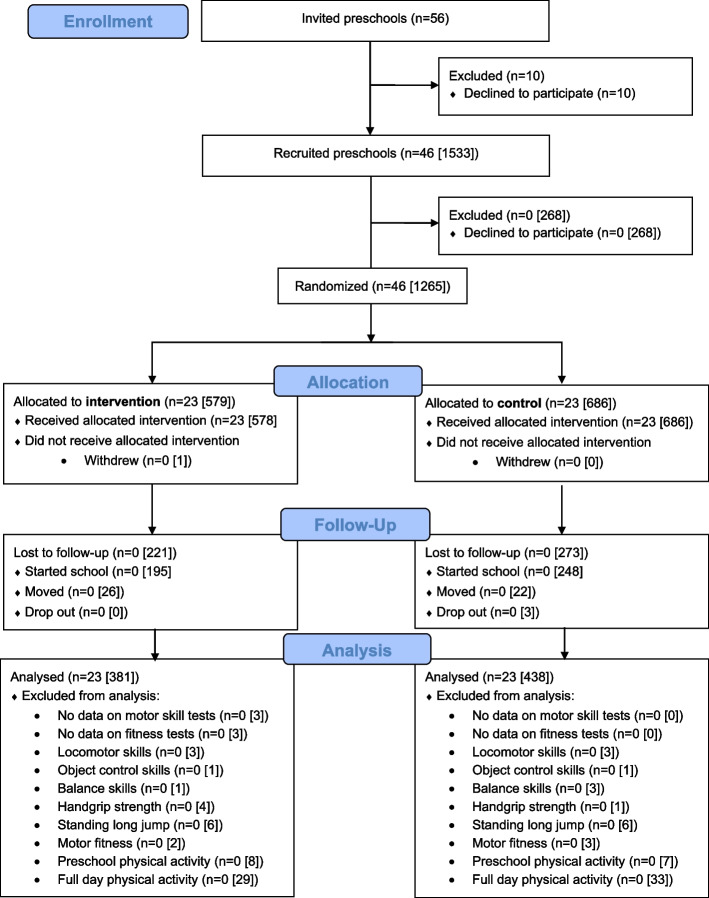
The flow of preschools and children through the study. All numbers are preschools [children]

Of 56 invited preschools, 46 agreed to participate (response rate 82%). Out of 1533 children from included preschools, 1265 consented via parent(s)s or guardian(s) (response rate 82%) (CONSORT checklist, Additional file 2). We included 819 children aged 3–4 years (53.5% boys) in the primary analyses having 7- and 18-month follow-up (Fig. 1), whereas children aged 5 years (*n* = 443) only having 7-month follow-up were included in secondary analyses [39]. From the 23 intervention preschools, a total of 77 directors and teachers participated in the professional development.

Included children provided valid data on at least one of the FMS or FIT outcomes and were excluded if they had no valid data for both FMS and FIT (*n* = 3). Table 1 shows included children’s baseline characteristics. Descriptive data for FMS, FIT, and PA at 7- and 18-month follow-ups can be found in additional files 3 and 4. Missing data were mainly due to children not wanting to participate in testing, had non-valid accelerometer data or had started school before the 18-month follow-up took place.

**Table 1:** Baseline characteristics for included children

	**Intervention**	**Control**
**Variable**	Mean (SD) or frequencies	Mean (SD) or frequencies
Age (years)	3.8 (0.6)	3.7 (0.6)
Sex (% boys)	55.1	52.1
BMI (Kg/m2)	16.3 (1.4)	16.4 (1.5)
Normal weight (%)	83.6	84.3
Overweight (%)	15.0	12.0
Obese (%)	1.4	3.6
*Parents*’*education level (%)*		
≤ Upper secondary school	26.4	26.4
University < 4 years	29.0	27.7
University ≥ 4 years +	44.5	45.9
*PA full day*		
Wear time (min/day)	761 (71)	757 (66)
Total PA (cpm)	647 (139)	667 (141)
SED (min/day)	537 (66)	533 (61)
LPA (min/day)	153 (22)	152 (21)
MPA (min/day)	37 (7)	37 (7)
VPA (min/day)	34 (9)	35 (10)
MVPA (min/day)	71 (16)	72 (16)
60 min/day MVPA (%)	76	78
*PA during preschool*		
Wear time (min/day)	433 (19)	426 (21)
Total PA (cpm)	751 (197)	790 (199)
SED (min/day)	284 (23)	278 (24)
LPA (min/day)	101 (16)	99 (14)
MPA (min/day)	25 (6)	25 (6)
VPA (min/day)	23 (8)	24 (8)
MVPA (min/day)	47 (13)	49 (13)
*FMS*		
Locomotor skills	8.9 (3.8)	8.8 (3.9)
Object control skills	6.9 (2.8)	6.3 (3.1)
Balance skills	8.4 (5.2)	7.0 (5.2)
*Physical fitness*		
Handgrip strength (kg)	7.2 (2.1)	6.8 (2.1)
Standing long jump (cm)	69.5 (21.2)	65.2 (24.6)
Motor fitness (sec)	19.4 (2.7)	19.7 (3.0)

*SD* Standard Deviation, *BMI* Body Mass Index. Weight status according to Cole et al. [58]. SES defined as highest educational level off child`s mother or father. PA guidelines defined by the Norwegian health directorate [11] and WHO [3]. PA intensity categories defined according to Evenson et al. [57]. FMS: Fundamental motor skill, sum score between 0 (lower skill level) and 22 (for object control skills) or 24 (for locomotion and balance skills). N (intervention): 381; FMS *n* = 324–346; FIT *n* = 312–351. N (control): 438; FMS *n* = 381–399; FIT *n* = 365–394. N (total): FMS *n* = 705–745; FIT n = 677–745

### Intervention adherence

Mean attendance to seminars and submissions of written work from staff in intervention preschools were 79%, and 13 out of 23 preschools attended to ≥ 80%. Thirteen out of 23 intervention preschools self-reported that integration was successful to a high or very high degree. Fourteen preschools were rated either “fair” or “good” in researchers᾽ assessment of intervention commitment and management.

### Primary analyses

In the primary analyses (Table 2) we found positive effects of the intervention on object control skills at 7 months (standardized ES = 0.17), and for locomotor skills at 18 months (ES = 0.21). For handgrip strength, we found a negative effect at 7 months (ES = -0.16). We found no significant effects for balance skills, standing long jump, or motor fitness (ESs = -0.11 to 0.05).

**Table 2:** Intervention effects for FMS and FIT measures at 7- and 18-month follow-up

			**7-month follow-up**			**18-month follow-up**		
	N	ICC	Estimate (95% CI)	ES	*p*	Estimate (95% CI)	ES	*p*
**Locomotor skills**	813	0.04	0.04 (-0.47–0.54)	0.01	0.880	0.81 (0.30–1.31)	**0.21**	**0.002**
**Object control skills**	817	0.04	0.51 (0.02–1.01)	**0.17**	**0.042**	0.17 (-0.33–0.67)	0.06	0.503
**Balance skills**	815	0.05	-0.58 (-1.25–0.10)	-0.11	0.096	0.06 (-0.61–0.74)	0.01	0.858
**Handgrip strength (kg)**	814	0.03	-0.34 (-0.57–-0.11)	**-0.16**	**0.003**	0.15 (-0.08–0.04)	0.07	0.196
**Standing long jump (cm)**	807	0.04	-1.95 (-4.52–0.62)	-0.08	0.136	-1.56 (-4.16–1.04)	-0.07	0.239
**Motor fitness (sec)**	814	0.04	0.14 (-0.15–0.43)	0.05	0.340	0.10 (-0.19–0.39)	0.04	0.497

*ICC* Intraclass correlation coefficient, *ES* Standardized effect size. FMS-outcomes were adjusted for FMS assessor. Statistical significance at p $\le$ 0.05 highlighted in bold

During preschool hours, we found a significant negative effect for SED at 7 months (standardized ES = -0.18) (Table 3). Further, we found significant positive effects for LPA (ES = 0.14) and MVPA (ES = 0.16) at 7 months. At 18 months, there were no significant effects for PA, except for a negative effect for LPA (ES = -0.15). A positive (non-significant) trend was evident for VPA at 7- (ES = 0.16) and 18-month follow-ups (ES = 0.15) during preschool hours. We found no significant effects for PA over the full day.

**Table 3:** Intervention effects for PA (full day and preschool hours) at 7- and 18-month follow-up

			**7-month follow-up**			**18-month follow-up**		
	N	ICC	Estimate (95% CI)	ES	*p*	Estimate (95% CI)	ES	*p*
**Preschool hours**								
Total PA (cpm)	804	0.08	15.41 (-19.40–50.22)	0.08	0.385	17.51 (-17.72–52.75)	0.09	0.330
SED (min/day)	804	0.11	-4.36 (-7.52–-1.21)	**-0.18**	**0.007**	0.96 (-2.21–4.12)	0.04	0.553
LPA (min/day)	804	0.14	2.08 (0.16–4.01)	**0.14**	**0.034**	-2.19 (-4.12–-0.25)	**-0.15**	**0.027**
MPA (min/day)	804	0.11	0.78 (0.03–1.59)	0.13	0.060	-0.16 (-0.98–0.65)	-0.03	0.697
VPA (min/day)	804	0.06	1.27 (-0.03–2.56)	0.16	0.055	1.16 (-0.14–2.46)	0.15	0.079
MVPA (min/day)	804	0.06	2.07 (0.21–3.93)	**0.16**	**0.029**	1.01 (-0.86–2.87)	0.08	0.290
**Full day**								
Total PA (cpm)	757	0.05	-2.55 (-29.00–23.90)	-0.02	0.850	16.48 (-9.91–42.88)	0.12	0.221
SED (min/day)	757	0.05	0.89 (-3.54–5.31)	0.01	0.695	1.17 (-3.25–5.59)	0.02	0.604
LPA (min/day)	757	0.07	-0.77 (-3.52–1.99)	-0.04	0.585	-2.15 (-4.90–0.61)	-0.10	0.126
MPA (min/day)	757	0.05	-0.33 (-1.35–0.68)	-0.05	0.519	-0.21 (-1.23–0.80)	-0.03	0.681
VPA (min/day)	757	0.02	-0.01 (-1.55–1.54)	0.00	0.995	0.99 (-0.55–2.53)	0.10	0.208
MVPA (min/day)	757	0.03	-0.26 (-2.59–2.07)	-0.02	0.824	0.83 (-1.49–3.16)	0.05	0.483

*ICC* Intraclass correlation coefficient, *ES* Standardized effect size; analyses were adjusted for accelerometer wear time. Statistical significance at *p* $\le$ 0.05 highlighted in bold

### Secondary analyses

We found significant moderating effects across sex, age, and baseline performance (Additional files 5–6), however, findings were characterized by low systematicity. Boys showed a significant positive effect in handgrip strength at 18 months (ES = 0.32), whereas girls showed a non-significant negative trend (ES = -0.16) (Additional file 5A). The lowest performing group for balance skills showed a significant positive effect at 18 months (ES = 0.67), whereas the best performing group showed a negative and non-significant trend (ES = -0.05) (Additional file 5B). Younger children showed a favorable negative effect on sedentary time at 7 months (ES = -0.18) compared to their older peers (ES = 0.05, non-significant) (Additional file 6C). No significant effects were found for moderation by baseline performance for PA (Additional file 6B) or moderation by age for FMS and FIT (Additional file 5C).

For FMS and FIT, results mainly align with the primary analysis for all three per-protocol analyses (Additional file 7a, 8a, and 9a), except for object control skills where effects were non-significant in all per-protocol analyses. However, positive effects for locomotor skills at 18 months, and negative effects for handgrip strength at 7 months were consistently found in all per-protocol analyses. For the researcher assessment of intervention implementation, handgrip strength showed a positive effect at 18 months (ES = 0.15). For PA analyses regarding attendance at seminars and completion of written work, and the researcher assessment of intervention implementation (Additional file 7b and 9b), results were similar to the primary analysis showing favorable effects during preschool hours for SED (ES = -0.17 to -0.29), LPA (ES = 0.10 to 0.26), and MVPA (ES = 0.18 to 0.22) at 7 months, and a negative effect for LPA at 18 months (ES = -0.19 to -0.22). Analyses of self-reported intervention integration (Additional file 8b) showed significant and favorable effects at 7- and 18-months follow-ups during preschools hours for SED (ES = -0.24 to -0.33) and all PA intensities (ES = 0.23 to 0.32) expect for LPA at 18 months. Furthermore, the same analysis over the full day showed a small, significant decrease in SED (ES = -0.13) and increases in all PA intensities (ES = 0.16 to 0.41) at 18 months.

We found weak but significant associations between change in PA and change in outcomes over 18 months, especially for higher PA intensities (Additional file 10). Change in VPA during preschool hours were positively associated with change in locomotor skills (β = 0.16), balance skills (β = 0.09), and standing long jump (β = 0.10). Change in MVPA were positively associated with change in locomotor skills (β = 0.16) and standing long jump (β = 0.10), and negatively associated with motor fitness (β = -0.10). Change in SED was negatively associated with standing long jump (β = -0.13). For PA over the full day, change in VPA and MVPA (β = 0.10–0.11) were positively associated with change in locomotor skills, and change in SED was negatively associated with standing long jump (β = -0.26).

## Discussion

The ACTNOW study aimed to test the effects of a pragmatic PA intervention for children delivered by preschool staff who participated in professional development in PA. The content of the professional development was designed to strengthen the staff’s competence and capacity in promoting PA and child development. Our findings showed small positive effects of the intervention on object control skills at 7 months and locomotor skills at 18 months. No positive effects were found for the FIT measures, except an unexpected negative effect for handgrip strength at 7 months. We found small, favorable effects for PA during preschool hours at 7 months, but these effects were not sustained at 18 months. Subgroup analyses revealed few systematic effects across all outcomes. Results from the per-protocol analyses were generally consistent with the primary results.

Our partly positive intervention effects for locomotor skills, object control skills, and PA align with findings in previous meta-analyses and systematic reviews showing beneficial effects of preschool PA interventions on FMS [9, 26–28] and objectively measured PA [63, 64]. Our null-finding for balance aligns with some previous studies [31, 65, 66] but contradicts others [67]. Of the relevant studies in the meta-analysis by Koolwijk et al. [28], most studies showed positive findings on one or several FMS outcomes, mainly on object control skills. However, the included studies had short-term follow-ups (≤ 9 months), small samples (n ≤ 162), and the FMS assessment tools varied between studies, reducing comparability [28, 68]. Moreover, most studies had rather structured PA programs, contrasting our more pragmatic and holistic intervention approach. Large differences in study characteristics were also evident across studies included in a meta-analysis [9], showing positive effects of interventions in preschool settings for both locomotor and object control skills. However, the authors graded most of the included studies to be of low quality, and the few high-quality studies of longer duration in which preschool staff were responsible for delivering the intervention, proved less effective. The lack of long-term effects in existing literature may suggest that FMS needs to be practiced and reinforced regularly [8], and the current study effect for locomotor skills at 18 months shows that this can be achieved through a pragmatic, staff-led intervention. Although we found mixed findings for FMS, the effects on preschool PA were significant and positive at 7 months, however not sustained at 18 months. The diminishing long-term effects may be explained by a lack of motivation and engagement among staff, limited transfer of knowledge or turnover of staff, or less follow-up of staff during year 2 of the intervention, which may have resulted in poor compliance to the intervention. However, timing of developmental trajectories in children may contribute explain the significant short-term effect in object control skills knowing that these develop later and may therefore not have emerged yet [6], thus stimulated through the professional development. However, the stimulation of already existing locomotor skills may have been efficacious in the longer-term. Per-protocol analyses showed positive effects for locomotor skills across all criteria, supporting the findings from the primary analysis. In contrast, results on object control skills were not significant in any of the per-protocol analyses, indicating that ball games and manipulation skills may have been less prioritized in some preschools and therefore not stimulated enough to be efficacious. Effects on PA in preschools with the highest levels of integration were also positive at 18 months both during preschool hours and over the full day; however, no effects were evident for object control or balance skills irrespective of the apparent PA increase, indicating that the amount, or types of PA may not be sufficient to create a long-term impact.

Few differences in effects between boys and girls in this study may be a result of focusing all children in the intervention, in contrast to previous literature showing that boys may benefit more from preschool than girls with regard to PA participation [69]. For FMS, our results differ from previous studies [70], showing that boys had larger effects than girls on object control skills. However, few studies have included moderation or sex-specific analyses [32]. The efficacy of a robust professional development ensuring that girls enjoy PA in the same way as boys is important and may counteract the gender differences in FMS [70]. In regard to the balance domain, we found that children with the poorest balance skills had the greatest effect, indicating that the intervention to some extent was efficacious in contributing to dampen health inequalities.

The (main) sample of 3–4-year-olds showed more favorable effects for SED, LPA, and MVPA during preschool hours compared to 5-year-olds at 7 months. However, the only significant difference between groups was for SED where the younger group were less sedentary. The smaller effects in the older group may be due to these children having a higher initial motor competence, thus capable of more physically active play than the younger children which need more support in their PA, resulting in reduced differences in PA across groups. Another explanation may be an increased focus on curricula and learning outcomes in preparing for school, thus making PA less prioritized.

For the fitness outcomes, our primary analyses showed no intervention effects on standing long jump or motor fitness, contrasting previous studies [30–33]. Wang et al. [33] showed positive effects on grip strength, standing long jump, and motor fitness following a multi-component intervention; however, their intervention program was structured and lasted only 16-weeks, thus limiting comparison with our study. It is not clear why we found different effects for locomotor skills and FIT measures for the standing long jump and running/motor fitness, for which assessments are rather similar. Likely, the difference may be attributable to the methodological differences in scoring of process- vs product-oriented assessments (i.e., qualitative vs quantitative outcomes, respectively) of similar motor skills. However, the difference may also lie in that the flexibility provided to teachers allowed them to focus on skills with less fitness components. Future research should monitor the activities chosen by preschool teachers to test this possibility. This may also explain the negative effect for handgrip strength at 7 months, which contrasts previous studies showing positive [32] or null effects [31]. Abe et al. [34] investigated whether PA interventions could improve handgrip strength in children and concluded that including specific gripping exercises may be effective, however, this was not a focus in the current intervention.

Interestingly, we did find a positive effect for handgrip strength at 18 months in the per-protocol analyses of researchers’ evaluation of the preschools’ commitment and management, which suggests results may be explained by the level of preschools᾽ intervention integration [36]. We found effect moderation by sex for handgrip strength at 18 months, where boys showed a positive effect, whereas girls had a negative effect. Boys often receive more encouragement and opportunities for PA than girls [71], possibly resulting in different engagement in grip strengthening activities (i.e., climbing or rough-and-tumble play) and shows that this may be a skill that needs to be specifically addressed to achieve favorable effects [34]. In the study by Mačak et al. [32] they found significant improvements for boys on standing long jump and motor fitness, contrasting the current study findings. The absence of certain effects in the current study indicate that preschool staff may not have gained sufficient competence to change a PA practice, and that there may be a need for teachers to broaden their repertoire of activities and pedagogical approaches (e.g., non-linear pedagogy) to stimulate a more nuanced development of children`s motor skills [72]. The content and amount of staff training, which often is low or not reported in previous studies [28, 31, 63], will likely impact both engagement in delivering an intervention, and results at the child level [37].

Current literature is shifting towards and recommending a more flexible intervention approach for early childhood development, as applying structured programs delivered by experts largely ignores the context as a critical factor for intervention effectiveness [38]. More than half of the intervention preschools reported that the intervention was successfully integrated into their everyday practices, supporting our findings for FMS and PA. This is also substantiated by our correlation analyses showing positive associations between change in PA and (several) FMS (and FIT) outcomes. Further, limited time for intervention implementation and competing priorities were reported by the intervention preschools and confirmed by researchers’ observations, partly due to staffing challenges and the Covid pandemic. These challenges are consistent with previously reported barriers to intervention implementation in preschool and school settings [73–75].

## Strengths and limitations

The main strengths of this study were the pragmatic approach of having teachers deliver the intervention, the cluster RCT-design, and the large sample size, allowing for detecting small to moderate effects at the child level. However, readers should keep in mind that we investigated secondary outcomes of the ACTNOW study, which may have increased the chance of type I errors.

The amount of professional development, the specified intervention components, and long-term follow-up of intervention preschools are additional strengths. It was novel to provide the in-depth training of teachers. As such, this study has great ecological validity, but it limits our ability to determine the exact dosage or type of activities implemented. This complicates comparison across studies, interpretation, and implications. Although researchers and test personnel were blinded for group allocation, a limitation was that some intervention preschools displayed ACTNOW-material during testing that revealed their allocation.

The study was conducted in a rural area in Western Norway. Although the sample size and response rate were high, generalizations should be cautious due to differences in preschool practices across contexts. The lack of a gold standard for assessing FMS [76], and the use of a modified FMS test battery [43] in the current study limits comparability with previous studies. Despite this, we regard including balance skills, which is less common, as a strength. Moreover, we regard the inclusion of both FMS and FIT measures a strength, broadly capturing different aspects of children's physical development. However, we did not include cardiorespiratory fitness among the FIT measures due to limited space in preschools. The use of other accelerometer cut points could potentially have affected our results, but we find any meaningful change in effects unlikely.

All preschool staff delivered the intervention. Thus, the competence and prerequisites to change a PA practice beyond those participating in the professional development might have been limited. Importantly, Covid-19 likely led to further challenges for implementation, given the reduced capacity to focus on the ACTNOW-intervention. Infection control guidelines recommended spending more time outdoors, which may have reduced group differences in MVPA [77].

## Conclusion

This study provides evidence of a short-term effect on object control skills, and a long-term effect on locomotor skills for young children following a PA intervention delivered by staff that participated in professional development. Children reduced their sedentary time and increased PA in the short-term, showing that enhancing staff competence on PA and knowledge of its relevance for child development can be effective for improving young children’s movement behavior. However, effects were mixed, and more research is needed to investigate both short- and long-term effects and sustainability of pragmatic PA interventions conducted in the preschool setting on children’s PA and physical development.

### Supplementary Information

Supplementary Material 1:  Additional file 1. Description of the ACTNOW intervention. Additional file 2. CONSORT checklist. Additional file 3. Physical activity descriptive table of included sample. Mean (SD) of baseline, 7-months, and 18-months physical activity levels. Additional file 4. FMS and FIT descriptive table of included sample. Mean (SD) scores for baseline, 7-months, and 18-months for fundamental motor skills and physical fitness. Additional file 5 A-C. Subgroup analyses of FMS and FIT. Secondary effects (group*time) on fundamental motor skills and physical fitness in subgroups A) sex, B) baseline performance, and C) age. Additional file 6 A-C. Subgroup analyses of physical activity. Secondary effects (group*time) on physical activity in subgroups A) sex, B) baseline performance, and C) age. Additional file 7 A-B. Per-protocol analyses of A) FMS and FIT, and B) PA, based on attendance and delivery of written assignments. Additional file 8 A-B. Per-protocol analyses of A) FMS and FIT, and B) PA, based on preschools᾽ evaluation of the extent to which ACTNOW became integrated in the everyday practice. Additional file 9 A-B. Per-protocol analyses of A) FMS and FIT, and B) PA, based on researchers᾽ experiences of overall commitment and management. Additional file 10. Regression coefficients of PA, FMS, and FIT change scores. Standardized regression coefficients of change scores (T3-T1) between PA, FMS, and FIT. Additional file 11. TIDieR Checklist.

## Data Availability

The datasets used and analysed during the current study are available from the corresponding author upon reasonable request.

## Abbreviations

BMI: Body mass index
cpm: Counts per minute
ES: Effect size
FIT: Physical fitness
FMS: Fundamental motor skills
LPA: Light physical activity
min: Minutes
MPA: Moderate physical activity
MVPA: Moderate-to-vigorous physical activity
PA: Physical activity
SD: Standard deviation
SED: Sedentary time
SES: Socioeconomic status
VPA: Vigorous physical activity
