# Supplementary material for: Effects of a staff-led multicomponent physical activity intervention on preschooler's fundamental motor skills and physical fitness: The ACTNOW cluster-randomized controlled trial
Source: Int J Behav Nutr Phys Act. 2024 Jul 3;21:69. doi: 10.1186/s12966-024-01616-4 (PMC11223439; doi:10.1186/s12966-024-01616-4)
Supplement: Supplementary file 1 — Supplementary Material 1: Additional file 1. Description of the ACTNOW intervention. Additional file 2. CONSORT checklist. Additional file 3. Physical activity descriptive table of included sample. Mean (SD) of baseline, 7-months, and 18-months physical activity levels. Additional file 4. FMS and FIT descriptive table of included sample. Mean (SD) scores for baseline, 7-months, and 18-months for fundamental motor skills and physical fitness. Additional file 5 A-C. Subgroup analyses of FMS and FIT. Secondary effects (group*time) on fundamental motor skills and physical fitness in subgroups A) sex, B) baseline performance, and C) age. Additional file 6 A-C. Subgroup analyses of physical activity. Secondary effects (group*time) on physical activity in subgroups A) sex, B) baseline performance, and C) age. Additional file 7 A-B. Per-protocol analyses of A) FMS and FIT, and B) PA, based on attendance and delivery of written assignments. Additional file 8 A-B. Per-protocol analyses of A) FMS and FIT, and B) PA, based on preschools᾽ evaluation of the extent to which ACTNOW became integrated in the everyday practice. Additional file 9 A-B. Per-protocol analyses of A) FMS and FIT, and B) PA, based on researchers᾽ experiences of overall commitment and management. Additional file 10. Regression coefficients of PA, FMS, and FIT change scores. Standardized regression coefficients of change scores (T3-T1) between PA, FMS, and FIT. Additional file 11. TIDieR Checklist. [file 12966_2024_1616_MOESM1_ESM.pdf]

Additional file 1

Description of the ACTNOW intervention

Table 1. Overview of the professional development (preschool level).

**Structure**	The professional development was structured as a 15-credit continuing education module at a Master's degree level delivered over 7 months. However, the staff chose whether they completed the exam and achieved credits or completed the professional development without credits. In total, 77 directors and teachers participated in the professional development, of which 16 of these achieved credits.<br><br>The professional development was structured as 6 days of face-to-face seminars (4 days on Campus and 2 days locally in each preschool), 2 webinars (4 hours in total) and 9 digital lectures (7 lectures of 20 min. each about the intervention content at child level, and 2 lectures of 1 hour each about the development work process at the preschool level), amounting to approximately *50 hours* in total. In addition, we had a one-day physical booster session during year 2 (12 months after kick-off). *Note. One of the face-to-face seminars in both waves had to be digital due to regulations during the Covid-pandemic.*
**Requirements for participation**	The preschool director and a minimum of one teacher from each classroom participated in the professional development.
**Aims/content**	The aims of the professional development were to increase the preschool directors' and teachers' competence regarding<br>1. the importance of physically active play and its relevance for child development, and how to integrate more physically active play (the core components) into the preschools practice; and<br>2. planning and implementation of interventions/changing practice, and thus facilitate the ACTNOW implementation process within the entire preschool and its staff.
**Process**	The professional development had three phases<br>**Phase 1: Setting the stage (2 months)**<br>The professional development started with an intensive 2-day face-to-face seminar. One part of the seminar focused on the intervention at the child level, with both practical and theoretical sessions regarding the four core components, as well as general topics on PA and physically active play, motor competence, cognitive development, and didactics. The other part of the seminar focused on the process of the development work among the preschool staff. The seminar was followed by a 1-day visit to each preschool by a member of the research group. This visit included a half-day observation of practice and a 2-hour staff meeting, planned by the preschools, including discussion of each preschool's specific PA practice, contextual factors, needs, resources etc. During this first phase the directors/teachers drafted a model on how to integrate the four core components into their daily practice, which was subject to feedback and discussion in an individual webinar with the same member from the research group that visited the preschool.<br><br>**Phase 2: Customize the intervention for sustainability (3 months)**<br>The second phase of the professional development focused on the implementation of each preschool's intervention model at the child level. Based on the initial experiences and discussions in Phase 1, each preschool model was revised and further developed to meet with the preschool practice and the prescribed intervention dose. During this second phase, 5 digital lectures (4x20 min. lectures about the intervention model and components at the child level, didactics, learning environment and teachers as role models, and 1x60 min. lecture about the developmental work process at the preschool level) was given to extend the lectures and discussions in Phase 1. One face-to-face seminar was arranged, where participants and the research group meet to share the preschools' experiences regarding the implementation process. During this second phase, staff

	from each classroom submitted a week-plan from each month, showing their implementation of physical activity in their daily schedule. Furthermore, the staff were encouraged to submit examples of activities that they developed and used, in which the research group processed and published in the toolbox, facilitating ideas sharing between the intervention preschools.<br><br>**Phase 3: Reflect on and evaluate the intervention (2 months)**<br>The third phase of the professional development focused on reflection and evaluation of the changed preschool practice regarding the implementation of the preschool's intervention model. Five digital lectures (3x20 min. lectures focusing on the intervention components and 1x60 min lecture on leading a developmental working process) and one 2 hours webinar (related to the developmental work) was given, in addition to a 1-day visit in each preschool, with a new half-day observation, followed by a joint reflection in the whole staff group about the developmental work and changed practices. The professional development ended with a full-day seminar of experience sharing among all intervention preschools, focusing on lessons learned and the way forward. Also, during this phase, staff from each classroom submitted one week-plan representing each month.

List of webinars:

1. Rationale for the intervention model (child level) (20 min lecture)
2. Didactics in physical activity, teaching principles (20 min lecture + task for reflection in staff)
3. Learning environment; motivation and mastery (20 min lecture + task for reflection)
4. Motor challenging physical activity (20 min lecture + encouragement to submit activities including object control skills)
5. Developmental work process; resistance to change and learning (60 min lecture + 4 reflection questions)
6. Developmental work process; leading/managing knowledge processes (60 min lecture)
7. Physically active learning (20 min lecture + development of activity integrating physical activity in the theme planned for the present period)
8. Cognitively engaging physically active play (20 min lecture)
9. The relationship between physical activity and motor skills (20 min lecture)

Description of the digital toolbox:

The content within the password protected digital toolbox was developed through cooperation between the research team and the staff in the intervention preschools. The toolbox consisted of different types of physical activities representing the four core components in the intervention level. We categorized the activities by using the colors and figures from the intervention model (i.e., activities that were cognitively engaging had a green template). All activities were presented with a picture, aims, procedure, possible variations, and equipment needed. Some activities also had tips to the staff. The toolbox also included four short texts and videos (2 minutes) explaining each of the four core components. The videos were of children playing games from the toolbox, representing each of the component.

The toolbox has been further developed after the project period and is now available in English from Aktiv i barnehagen.

Description of the portable movement and learning equipment:

Each classroom within the intervention preschool received a bag with movement and learning equipment during the first face-to-face seminar. The equipment bag included 19 balls of different sizes, weights and materials, 10 jumping ropes, 8 hula-hoops, 3 large dices from 1-6, 8 sensory slices, 10 cones and 5 hurdle sticks and one mat (all shown in picture). We supplied with more balls, hula-hoops, and dices from 1-12 during the face-to-face seminar after 12 months.

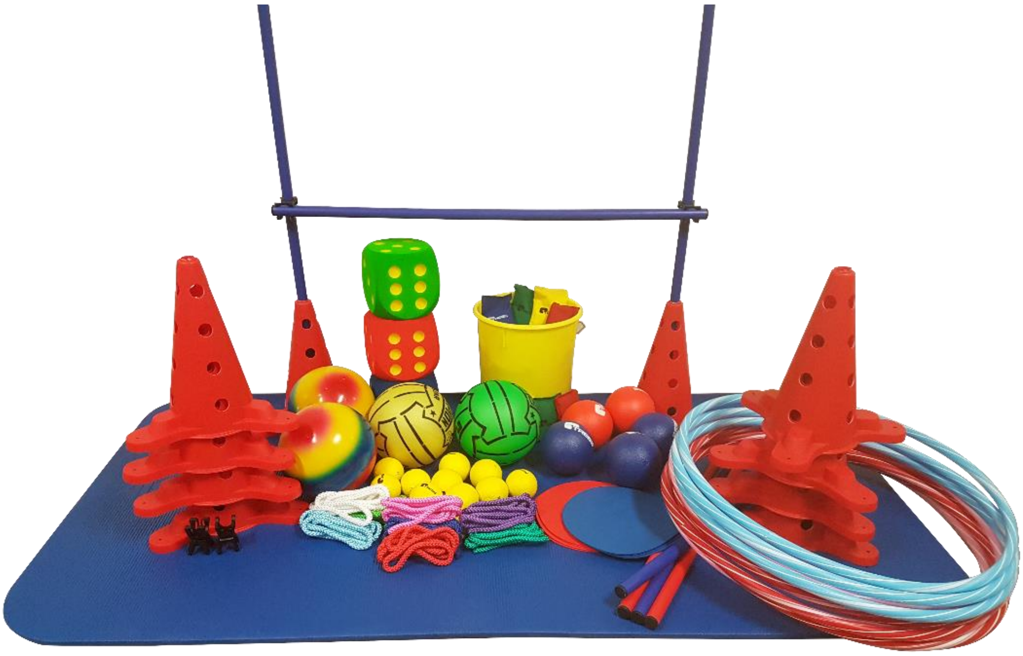

Timeline of professional developemnt.

	Year 1							Year 2
	Nov	Dec	Jan	Feb	Mar	Apr	May	Nov
Face-to-face seminar	xx		x				x	x
Visits to preschools	x					x		
Digital lectures			x	x	x	x	x	x
Webinar		x			x			
Submission of written work		x	x	x	x	x	x	

Additional file 2

CONSORT checklist

Tables

Table 1| **CONSORT 2010 checklist of information to include when reporting a cluster randomised trial**

Section/topic and item No	Standard checklist item	Extension for cluster designs	Page No*
**Title and abstract**			
1a	Identification as a randomised trial in the title	Identification as a cluster randomised trial in the title	p.1
1b	Structured summary of trial design, methods, results, and conclusions (for specific guidance see CONSORT for abstracts)[11 12]	See table 2	p.2
**Introduction**			
Background and objectives:			
2a	Scientific background and explanation of rationale	Rationale for using a cluster design	p. 3-4
2b	Specific objectives or hypotheses	Whether objectives pertain to the cluster level, the individual participant level, or both	p.4
**Methods**			
Trial design:			
3a	Description of trial design (such as parallel, factorial) including allocation ratio	Definition of cluster and description of how the design features apply to the clusters	p. 4
3b	Important changes to methods after trial commencement (such as eligibility criteria), with reasons		N/A
Participants:			
4a	Eligibility criteria for participants	Eligibility criteria for clusters	p. 4
4b	Settings and locations where the data were collected		p. 4, 5-6
Interventions:			
5	The interventions for each group with sufficient details to allow replication, including how and when they were actually administered	Whether interventions pertain to the cluster level, the individual participant level, or both	p. 5
Outcomes:			
6a	Completely defined prespecified primary and secondary outcome measures, including how and when they were assessed	Whether outcome measures pertain to the cluster level, the individual participant level, or both	p. 5-8
6b	Any changes to trial outcomes after the trial commenced, with reasons		N/A
Sample size:			
7a	How sample size was determined	Method of calculation, number of clusters(s) (and whether equal or unequal cluster sizes are assumed), cluster size, a coefficient of intracluster correlation (ICC or *k*), and an indication of its uncertainty	p. 4 & ref to protocol paper
7b	When applicable, explanation of any interim analyses and stopping guidelines		N/A
**Randomisation**			
Sequence generation:			
8a	Method used to generate the random allocation sequence		p. 4
8b	Type of randomisation; details of any restriction (such as blocking and block size)	Details of stratification or matching if used	p. 4
Allocation concealment mechanism:			
9	Mechanism used to implement the random allocation sequence (such as sequentially numbered containers), describing any steps taken to conceal the sequence until interventions were assigned	Specification that allocation was based on clusters rather than individuals and whether allocation concealment (if any) was at the cluster level, the individual participant level, or both	p. 4
Implementation:			
10	Who generated the random allocation sequence, who enrolled participants, and who assigned participants to interventions	Replaced by 10a, 10b, and 10c	
10a		Who generated the random allocation sequence, who enrolled clusters, and who assigned clusters to interventions	p. 4

BMJ: first published as 10.1136/bmj.e5661 on 4 September 2012. Downloaded from http://www.bmj.com/ on 18 August 2023 at Hogskulen pa Vestlandet. Protected by copyright.

Table 1 (continued)

Section/topic and item No	Standard checklist item	Extension for cluster designs	Page No*
10b		Mechanism by which individual participants were included in clusters for the purposes of the trial (such as complete enumeration, random sampling)	p. 4
10c		From whom consent was sought (representatives of the cluster, or individual cluster members, or both) and whether consent was sought before or after randomisation	p. 4
Blinding:			
11a	If done, who was blinded after assignment to interventions (for example, participants, care providers, those assessing outcomes) and how		p. 4
11b	If relevant, description of the similarity of interventions		N/A
Statistical methods:			
12a	Statistical methods used to compare groups for primary and secondary outcomes	How clustering was taken into account	p. 8
12b	Methods for additional analyses, such as subgroup analyses and adjusted analyses		p. 8
**Results**			
Participant flow (a diagram is strongly recommended):			
13a	For each group, the numbers of participants who were randomly assigned, received intended treatment, and were analysed for the primary outcome	For each group, the numbers of clusters that were randomly assigned, received intended treatment, and were analysed for the primary outcome	p. 9 (flow chart)
13b	For each group, losses and exclusions after randomisation, together with reasons	For each group, losses and exclusions for both clusters and individual cluster members	p. 9 (flow chart)
Recruitment:			
14a	Dates defining the periods of recruitment and follow-up		p. 4
14b	Why the trial ended or was stopped		N/A
Baseline data:			
15	A table showing baseline demographic and clinical characteristics for each group	Baseline characteristics for the individual and cluster levels as applicable for each group	p. 9-10 and additional files
Numbers analysed:			
16	For each group, number of participants (denominator) included in each analysis and whether the analysis was by original assigned groups	For each group, number of clusters included in each analysis	p. 10-11 and additional files
Outcomes and estimation:			
17a	For each primary and secondary outcome, results for each group, and the estimated effect size and its precision (such as 95% confidence interval)	Results at the individual or cluster level as applicable and a coefficient of intracluster correlation (ICC or *k*) for each primary outcome	p.10-11 and additional files
17b	For binary outcomes, presentation of both absolute and relative effect sizes is recommended		N/A
Ancillary analyses:			
18	Results of any other analyses performed, including subgroup analyses and adjusted analyses, distinguishing prespecified from exploratory		p. 10-11 and additional files
Harms:			
19	All important harms or unintended effects in each group (for specific guidance see CONSORT for harms106)		N/A
**Discussion**			
Limitations:			
20	Trial limitations, addressing sources of potential bias, imprecision, and, if relevant, multiplicity of analyses		p. 15
Generalisability:			
21	Generalisability (external validity, applicability) of the trial findings	Generalisability to clusters and/or individual participants (as relevant)	p. 15

BMJ: first published as 10.1136/bmj.e5661 on 4 September 2012. Downloaded from http://www.bmj.com/ on 18 August 2023 at Hogskulen pa Vestlandet. Protected by copyright.

Table 1 (continued)

Section/topic and item No	Standard checklist item	Extension for cluster designs	Page No*
Interpretation:			
22	Interpretation consistent with results, balancing benefits and harms, and considering other relevant evidence		p. 12-16
**Other information**			
Registration:			
23	Registration number and name of trial registry		p. 2
Protocol:			
24	Where the full trial protocol can be accessed, if available	https://www.ncbi.nlm.nih.gov/pmc/articles/PMC7350704/pdf/fpsyg-11-01382.pdf	
Funding:			
25	Sources of funding and other support (such as supply of drugs), role of funders		p. 17

*Page numbers optional depending on journal requirements.

BMJ: first published as 10.1136/bmj.e5661 on 4 September 2012. Downloaded from http://www.bmj.com/ on 18 August 2023 at Hogskulen pa Vestlandet. Protected by copyright.

For personal use only: See rights and reprints http://www.bmj.com/permissions Subscribe: http://www.bmj.com/subscribe

Table 2| **Extension of CONSORT for abstracts11 12 to reports of cluster randomised trials**

Item	Standard checklist item	Extension for cluster trials	
Title	Identification of study as randomised	Identification of study as cluster randomised	p. 2
Trial design	Description of the trial design (for example, parallel, cluster, non-inferiority)		p. 2
Methods:			
Participants	Eligibility criteria for participants and the settings where the data were collected	Eligibility criteria for clusters	
Interventions	Interventions intended for each group		p. 2
Objective	Specific objective or hypothesis	Whether objective or hypothesis pertains to the cluster level, the individual participant level, or both	p. 2
Outcome	Clearly defined primary outcome for this report	Whether the primary outcome pertains to the cluster level, the individual participant level or both	p. 2
Randomisation	How participants were allocated to interventions	How clusters were allocated to interventions	p. 2
Blinding (masking)	Whether or not participants, care givers, and those assessing the outcomes were blinded to group assignment	(p. 4)	
Results:			
Numbers randomised	Number of participants randomised to each group	Number of clusters randomised to each group	p. 2
Recruitment	Trial status*		
Numbers analysed	Number of participants analysed in each group	Number of clusters analysed in each group	
Outcome	For the primary outcome, a result for each group and the estimated effect size and its precision	Results at the cluster or individual level as applicable for each primary outcome	p. 2
Harms	Important adverse events or side effects		
Conclusions	General interpretation of the results		p. 2
Trial registration	Registration number and name of trial register		p. 2
Funding	Source of funding		p. 2

*Relevant to conference abstracts.

BMJ: first published as 10.1136/bmj.e5661 on 4 September 2012. Downloaded from http://www.bmj.com/ on 18 August 2023 at Hogskulen pa Vestlandet. Protected by copyright.

Additional file 3. Physical activity descriptive table of included sample. Mean (SD) of baseline, 7-months, and 18-months physical activity levels.

		Intervention			Control		
		**Baseline**	**7-months**	**18-months**	**Baseline**	**7-months**	**18-months**
**Preschool**	**n**	338	320	314	395	377	364
Total PA (cpm)		751 (197)	902 (232)	888 (250)	790 (199)	914 (260)	898 (233)
SED (min/day)		284 (23)	267 (27)	279 (28)	278 (24)	272 (25)	277 (22)
LPA (min/day)		101 (16)	106 (15)	98 (16)	99 (14)	105 (16)	100 (14)
MPA (min/day)		25 (6)	27 (7)	28 (7)	25 (6)	27 (6)	28 (6)
VPA (min/day)		23 (8)	28 (9)	28 (10)	24 (8)	28 (9)	28 (9)
MVPA (min/day)		47 (13)	55 (14)	56 (15)	49 (13)	55 (14)	57 (13)
**Full day**	**n**	317	271	270	348	307	317
Total PA (cpm)		647 (139)	779 (194)	752 (188)	667 (141)	795 (200)	741 (166)
SED (min/day)		537 (66)	517 (73)	538 (69)	533 (61)	519 (63)	542 (68)
LPA (min/day)		153 (22)	157 (22)	148 (20)	152 (21)	157 (21)	149 (21)
MPA (min/day)		37 (7)	39 (8)	40 (8)	37 (7)	40 (8)	40 (8)
VPA (min/day)		34 (9)	41 (12)	42 (12)	35 (10)	42 (12)	41 (11)
MVPA (min/day)		71 (16)	80 (18)	82 (18)	72 (16)	82 (18)	81 (16)

Additional file 4. FMS and FIT descriptive table of included sample. Mean (SD) scores for baseline, 7-months, and 18-months for fundamental motor skills and physical fitness.

	Intervention			Control		
	**Baseline**	**7-months**	**18-months**	**Baseline**	**7-months**	**18-months**
Locomotor skills	8.9 (3.8)	11.7 (4.1)	14.1 (3.8)	8.8 (3.9)	11.6 (4)	13.3 (3.9)
Object control skills	6.9 (2.8)	8.8 (3.5)	10.2 (4.1)	6.3 (3.1)	7.9 (3.3)	9.7 (3.7)
Balance skills	8.4 (5.2)	10.1 (5.1)	13.3 (4.9)	7 (5.2)	9.8 (5.4)	12.3 (4.9)
Handgrip strength (kg)	7.2 (2.1)	8.3 (2.3)	10.0 (2.4)	6.8 (2.1)	8.4 (2.4)	9.7 (2.3)
Standing long jump (cm)	69.5 (21.2)	81.9 (21.7)	95.4 (18.3)	65.2 (24.6)	81.5 (21.4)	95.0 (18.8)
Motor fitness (sec)	19.4 (2.7)	17.7 (2)	16.6 (2.2)	19.7 (3)	17.7 (2.3)	16.7 (2)

N intervention: FMS 324-350; FIT 312-360. N Control: FMS 381-407; FIT 365-418

Additional file 5 A-C. Subgroup analyses of FMS and FIT. Secondary effects (group*time) on fundamental motor skills and physical fitness in subgroups A) sex, B) baseline performance, and C) age.

A) Sex

				**7-month follow-up**							**18-month follow-up**						
							**95% CI**		**83.4% CI**					**95% CI**		**83.4% CI**	
	**Sex**	**N**	**ICC**	**Estimate**	**ES**	***p***	**Lower**	**Upper**	**Lower**	**Upper**	**Estimate**	**ES**	***p***	**Lower**	**Upper**	**Lower**	**Upper**
Locomotor skills	Boys	436	0.05	-0.06	-0.02	0.872	-0.74	0.63	-0.54	0.43	0.85	0.32	0.014*	0.17	1.54	0.37	1.34
	Girls	377	0.02	0.12	0.05	0.730	-0.58	0.82	-0.37	0.62	0.72	0.27	0.044*	0.02	1.42	0.22	1.22
Object control skills	Boys	437	0.05	0.53	0.25	0.130	-0.16	1.21	0.04	1.01	-0.11	-0.05	0.762	-0.79	0.58	-0.59	0.38
	Girls	380	0.04	0.61	0.29	0.071	-0.05	1.27	0.14	1.08	0.56	0.27	0.098	-0.10	1.22	0.09	1.03
Balance skills	Boys	435	0.03	-0.45	-0.12	0.335	-1.36	0.46	-1.09	0.20	0.45	0.12	0.325	-0.45	1.35	-0.19	1.09
	Girls	380	0.04	-0.38	-0.10	0.429	-1.31	0.56	-1.03	0.28	-0.01	0.00	0.982	-0.94	0.92	-0.67	0.65
Handgrip strength	Boys	434	0.06	-0.22	-0.15	0.154	-0.53	0.08	-0.44	-0.01	0.47	0.32	0.003*	0.16	0.78	**0.25**	**0.69**
	Girls	380	0.01	-0.49	-0.33	0.004*	-0.82	-0.16	-0.72	-0.26	-0.23	-0.16	0.169	-0.56	0.09	**-0.47**	**0.00**
Standing long jump	Boys	430	0.00	-1.86	-0.11	0.312	-5.45	1.74	-4.40	0.69	-2.05	-0.13	0.271	-5.45	1.74	-4.63	0.53
	Girls	377	0.00	-1.88	-0.11	0.302	-5.44	1.69	-4.40	0.65	-0.89	-0.05	0.628	-3.44	1.66	-3.44	1.66
Motor fitness	Boys	435	0.04	0.18	0.09	0.340	-0.19	0.56	-0.08	0.45	0.10	0.05	0.593	-0.17	0.37	-0.16	0.37
	Girls	379	0.05	0.05	0.03	0.801	-0.37	0.48	-0.25	0.35	0.07	0.03	0.765	-0.36	0.49	-0.24	0.37

ICC=Intraclass correlation coefficient; ES=effect size. FMS-outcomes were adjusted for FMS assessor. Significant differences between groups are highlighted in bold. *Significant differences within groups at $p \leq 0.05$.

B) Baseline performance

				7-month follow-up							18-month follow-up						
							95% CI		83.4% CI					95% CI		83.4% CI	
	Group	N	ICC	Estimate	ES	*p*	Lower	Upper	Lower	Upper	Estimate	ES	*p*	Lower	Upper	Lower	Upper
Locomotor skills	Group 1	359	0.03	0.06	0.04	0.853	-0.56	0.68	-0.38	0.50	0.46	0.29	0.147	-0.16	1.09	0.02	0.91
	Group 2	353	0.01	0.04	0.02	0.913	-0.62	0.69	-0.43	0.50	1.13	0.72	<0.001*	0.48	1.79	0.67	1.60
Object control skills	Group 1	409	0.05	0.70	0.53	0.027*	0.08	1.32	0.26	1.14	0.17	0.13	0.593	-0.45	0.79	-0.27	0.61
	Group 2	336	0.03	0.51	0.38	0.143	-0.17	1.18	0.03	0.98	0.23	0.17	0.517	-0.46	0.91	-0.26	0.71
Balance skills	Group 1	363	0.04	-0.52	-0.26	0.254	-1.40	0.37	-1.14	0.11	1.33	0.67	0.003*	0.45	2.22	**0.71**	**1.96**
	Group 2	342	0.04	0.11	0.06	0.783	-0.68	0.89	-0.44	0.66	-0.09	-0.05	0.822	-0.88	0.70	**-0.65**	**0.47**
Handgrip strength	Group 1	376	0.05	-0.15	-0.17	0.322	-0.46	0.15	-0.37	0.06	0.33	0.38	0.033*	0.03	0.64	0.12	0.55
	Group 2	369	0.04	-0.46	-0.52	0.004*	-0.77	-0.15	-0.68	-0.24	0.10	0.11	0.548	-0.22	0.41	-0.13	0.32
Standing long jump	Group 1	339	0.00	-0.23	-0.02	0.899	-3.78	3.32	-2.74	2.28	-0.61	-0.06	0.743	-4.26	3.04	-3.19	1.98
	Group 2	338	0.00	-1.63	-0.17	0.266	-4.51	1.25	-3.67	0.41	0.38	0.04	0.802	-2.55	3.30	-1.70	2.45
Motor fitness	Group 1	355	0.01	0.01	0.00	0.975	-0.40	0.41	-0.28	0.29	0.01	0.01	0.972	-0.40	0.41	-0.28	0.29
	Group 2	357	0.02	0.16	0.12	0.242	-0.11	-0.43	-0.03	0.35	0.04	0.03	0.776	-0.23	0.31	-0.15	0.23

ICC=Intraclass correlation coefficient; ES=effect size. FMS-outcomes were adjusted for FMS assessor. Significant differences between groups are highlighted in bold.
*Significant differences within groups at p≤0.05.

C) Age

				7-month follow-up						
							95% CI		83.4% CI	
	**Age**	**N**	**ICC**	**Estimate**	**ES**	***p***	**Lower**	**Upper**	**Lower**	**Upper**
Locomotor skills	3-4 yr	813	0.04	0.04	0.01	0.880	-0.47	0.54	-0.32	0.40
	5 yr	438	0.03	0.05	0.01	0.874	-0.60	0.71	-0.41	0.52
Object control skills	3-4 yr	817	0.04	0.51	0.17	0.042*	0.02	1.01	0.16	0.86
	5 yr	438	0.04	0.85	0.22	0.014*	0.17	1.52	0.37	1.32
Balance skills	3-4 yr	815	0.05	-0.58	-0.11	0.096	-1.25	0.10	-1.05	-0.10
	5 yr	438	0.03	-1.10	-0.24	0.006*	-1.88	-0.31	-1.65	-0.54
Handgrip strength	3-4 yr	814	0.03	-0.34	-0.16	0.003*	-0.57	-0.11	-0.50	-0.18
	5 yr	439	0.08	-0.11	-0.05	0.477	-0.42	0.20	-0.33	0.11
Standing long jump	3-4 yr	807	0.04	-1.95	-0.08	0.136	-4.52	0.62	-3.77	-0.13
	5 yr	435	0.05	-0.15	-0.01	0.910	-2.77	2.47	-2.01	1.70
Motor fitness	3-4 yr	814	0.04	0.14	0.05	0.340	-0.15	0.43	-0.06	0.34
	5 yr	437	0.09	0.18	0.10	0.158	-0.07	0.44	0.00	0.36

ICC=Intraclass correlation coefficient; ES=effect size. FMS-outcomes were adjusted for FMS assessor. Significant differences between groups are highlighted in bold. *Significant differences within groups at $p \leq 0.05$.

Additional file 6 A-C. Subgroup analyses of physical activity. Secondary effects (group*time) on physical activity in subgroups A) sex, B) baseline performance, and C) age.

A) Sex

				**7-month follow-up**							**18-month follow-up**						
							**95% CI**		**83.4% CI**					**95% CI**		**83.4% CI**	
	**Sex**	**N**	**ICC**	**Estimate**	**ES**	***p***	**Lower**	**Upper**	**Lower**	**Upper**	**Estimate**	**ES**	***p***	**Lower**	**Upper**	**Lower**	**Upper**
**Preschool**																	
Total PA	Boys	430	0.09	16.88	0.12	0.465	-28.41	62.17	-15.18	48.94	24.84	0.18	0.286	-20.87	70.56	-7.52	57.21
	Girls	374	0.09	3.42	0.02	0.896	-47.83	54.68	-32.85	39.69	-0.94	-0.01	0.972	-52.96	51.09	-37.75	35.88
SED	Boys	430	0.10	-3.95	-0.25	0.068	-8.18	0.29	-6.94	-0.95	1.37	0.09	0.527	-2.88	5.61	-1.64	4.37
	Girls	374	0.15	-4.16	-0.26	0.069	-8.64	0.33	-7.33	-0.98	1.20	0.07	0.602	-3.31	5.71	-1.99	4.39
LPA	Boys	430	0.14	1.99	0.20	0.123	-0.54	4.53	0.20	3.79	-2.82	-0.28	0.029*	-5.36	-0.28	-4.62	-1.03
	Girls	374	0.12	2.08	0.21	0.138	-0.67	4.84	0.13	4.04	-1.58	-0.16	0.263	-4.36	1.19	-3.54	0.38
MPA	Boys	430	0.09	0.83	0.21	0.151	-0.30	1.96	0.03	1.63	-0.14	-0.04	0.808	-1.28	1.00	-0.94	0.66
	Girls	374	0.15	0.52	0.13	0.356	-0.58	1.62	-0.26	1.30	-0.40	-0.10	0.477	-1.51	0.71	-1.18	0.38
VPA	Boys	430	0.07	1.05	0.19	0.239	-0.70	2.79	-0.19	2.28	1.52	0.27	0.088	-0.23	3.27	0.28	2.76
	Girls	374	0.07	1.09	0.19	0.248	-0.76	2.95	-0.22	2.41	0.32	0.06	0.734	-1.55	2.19	-1.00	1.65
MVPA	Boys	430	0.06	1.86	0.21	0.154	-0.70	4.43	0.05	3.68	1.34	0.15	0.308	-1.23	3.90	-0.48	3.15
	Girls	374	0.10	1.79	0.20	0.175	-0.80	4.39	-0.04	3.63	0.10	0.01	0.942	-2.51	2.71	-1.75	1.94
**Full day**																	
Total PA	Boys	405	0.04	3.50	0.04	0.846	-31.85	38.85	-21.52	28.51	4.46	0.05	0.804	-30.80	39.72	-20.49	29.42
	Girls	352	0.08	-10.74	-0.11	0.581	-48.97	27.50	-37.79	16.32	28.75	0.29	0.139	-9.34	66.84	1.80	55.70
SED	Boys	405	0.05	-0.55	-0.01	0.859	-6.65	5.54	-4.86	3.76	4.84	0.11	0.119	-1.24	10.93	0.54	9.15
	Girls	352	0.07	3.22	0.07	0.299	-2.86	9.30	-1.08	7.52	-3.10	-0.07	0.316	-9.15	2.96	-7.38	1.19
LPA	Boys	405	0.08	-0.24	-0.02	0.900	-3.96	3.48	-2.87	2.39	-4.55	-0.32	0.017*	-8.26	-0.83	**-7.18**	**-1.92**
	Girls	352	0.05	-1.59	-0.11	0.417	-5.43	2.25	-4.31	1.13	1.03	0.07	0.597	-2.79	4.86	**-1.68**	**3.74**
MPA	Boys	405	0.05	0.12	0.03	0.868	-1.32	1.57	-0.90	1.15	-0.96	-0.20	0.191	-2.41	0.48	-1.98	0.06
	Girls	352	0.09	-1.06	-0.22	0.121	-2.40	0.28	-2.01	-0.11	0.55	0.11	0.416	-0.78	1.89	-0.39	1.50
VPA	Boys	405	0.02	0.53	0.08	0.628	-1.61	2.67	-0.99	2.05	0.54	0.08	0.623	-1.60	2.68	-0.98	2.05
	Girls	352	0.05	-0.72	-0.11	0.522	-2.91	1.48	-2.27	0.84	1.44	0.22	0.197	-0.75	3.62	-0.11	2.98
MVPA	Boys	405	0.03	0.70	0.06	0.677	-2.58	3.97	-1.62	3.01	-0.42	-0.04	0.800	-3.69	2.85	-2.73	1.89
	Girls	352	0.07	-1.66	-0.15	0.308	-4.86	1.54	-3.92	0.60	2.09	0.19	0.199	-1.10	5.27	-0.17	4.34

ICC=Intraclass correlation coefficient; ES=effect size. Analyses were adjusted for accelerometer wear time. Significant differences between groups are highlighted in bold. *Significant differences within groups at $p \leq 0.05$.

B) Baseline performance

				7-month follow-up							18-month follow-up						
							95% CI		83.4% CI					95% CI		83.4% CI	
	**Group**	**N**	**ICC**	**Estimate**	**ES**	***p***	**Lower**	**Upper**	**Lower**	**Upper**	**Estimate**	**ES**	***p***	**Lower**	**Upper**	**Lower**	**Upper**
**Preschool**																	
Total PA	Group 1	387	0.06	5.05	0.05	0.798	-33.70	43.80	-22.36	32.47	11.59	0.13	0.562	-27.67	50.84	-15.93	39.10
	Group 2	389	0.09	17.21	0.19	0.513	-34.38	68.80	-19.30	53.72	7.21	0.08	0.788	-45.33	59.74	-29.97	44.39
SED	Group 1	388	0.13	-5.41	-0.55	0.014*	-9.72	-1.09	-8.46	-2.35	1.81	0.18	0.412	-2.52	6.14	-1.25	4.88
	Group 2	388	0.10	-3.47	-0.35	0.095	-7.54	0.61	-6.35	-0.58	0.63	0.06	0.764	-3.51	4.77	-2.30	3.56
LPA	Group 1	387	0.13	2.76	0.44	0.035*	0.19	5.34	0.94	4.58	-1.29	-0.20	0.321	-3.84	1.26	-3.10	0.51
	Group 2	389	0.18	0.70	0.11	0.591	-1.86	3.26	-1.11	2.51	-2.94	-0.46	0.027*	-5.55	-0.33	-4.78	-1.09
MPA	Group 1	388	0.09	-0.20	-0.08	0.694	-1.19	0.80	-0.90	0.50	-0.92	-0.37	0.068	-1.90	0.07	-1.61	-0.22
	Group 2	388	0.14	1.06	0.43	0.067	-0.08	2.20	0.26	1.87	0.17	0.07	0.776	-1.00	1.34	-0.66	1.00
VPA	Group 1	387	0.05	0.82	0.23	0.292	-0.71	2.34	-0.26	1.90	1.36	0.38	0.082	-0.17	2.89	0.27	2.44
	Group 2	389	0.09	0.80	0.23	0.402	-1.07	2.67	-0.53	2.12	-0.12	-0.03	0.900	-2.02	1.78	-1.46	1.22
MVPA	Group 1	388	0.06	1.03	0.19	0.362	-1.19	3.25	-0.54	2.60	-0.21	-0.04	0.854	-2.42	2.00	-1.77	1.36
	Group 2	388	0.09	1.59	0.29	0.241	-1.07	4.25	-0.29	3.47	0.91	0.16	0.509	-1.80	3.63	-1.01	2.83
**Full day**																	
Total PA	Group 1	373	0.05	3.94	0.07	0.803	-27.09	34.96	-18.01	25.89	22.33	0.37	0.159	-8.78	53.43	0.32	44.33
	Group 2	361	0.02	-3.02	-0.05	0.873	-40.21	34.18	-29.32	23.28	12.38	0.20	0.507	-24.30	49.05	-13.55	38.30
SED	Group 1	349	0.03	-1.27	-0.05	0.701	-7.78	5.23	-5.87	3.33	-1.20	-0.05	0.706	-7.46	5.06	-5.62	3.22
	Group 2	385	0.04	2.70	0.11	0.355	-3.03	8.43	-1.36	6.76	3.09	0.12	0.304	-2.81	8.98	-1.08	7.26
LPA	Group 1	359	0.08	0.10	0.01	0.956	-3.49	3.69	-2.44	2.64	0.57	0.06	0.755	-3.01	4.15	-1.97	3.11
	Group 2	375	0.06	-1.17	-0.13	0.514	-4.69	2.35	-3.66	1.32	-4.04	-0.45	0.025	-7.56	-0.52	-6.53	-1.55
MPA	Group 1	360	0.02	-0.63	-0.20	0.304	-1.84	0.58	-1.49	0.22	-0.22	-0.07	0.727	-1.43	1.00	-1.07	0.64
	Group 2	374	0.06	-0.16	-0.05	0.824	-1.53	1.22	-1.13	0.82	-0.36	-0.11	0.606	-1.72	1.01	-1.33	0.61
VPA	Group 1	364	0.01	0.45	0.11	0.629	-1.39	2.29	-0.85	1.75	1.12	0.27	0.232	-0.72	2.95	-0.18	2.41
	Group 2	370	0.04	-0.83	-0.20	0.450	-3.00	1.33	-2.36	0.70	0.85	0.21	0.437	-1.30	3.00	-0.67	2.37
MVPA	Group 1	362	0.02	-0.83	-0.12	0.563	-3.63	1.98	-2.81	1.16	1.32	0.19	0.353	-1.48	4.13	-0.66	3.30
	Group 2	372	0.04	0.09	0.01	0.955	-3.10	3.28	-2.16	2.34	0.34	0.05	0.834	-2.83	3.50	-1.90	2.58

ICC=Intraclass correlation coefficient; ES=effect size. Analyses were adjusted for accelerometer wear time. Significant differences between groups are highlighted in bold. *Significant differences within groups at $p \leq 0.05$.

C) Age

				7-month follow-up						
							95% CI		83.4% CI	
	Age	N	ICC	Estimate	ES	*p*	Lower	Upper	Lower	Upper
**Preschool**										
Total PA	3-4 yr.	804	0.08	15.41	0.08	0.385	-19.40	50.22	-9.28	40.10
	5 yr.	431	0.10	-21.88	-0.10	0.408	-73.78	30.03	-58.58	14.83
SED	3-4 yr.	804	0.11	-4.36	-0.18	0.007*	-7.52	-1.21	**-6.60**	**-2.13**
	5 yr.	431	0.09	1.25	0.05	0.543	-2.78	5.28	**-1.60**	**4.10**
LPA	3-4 yr.	804	0.14	2.08	0.14	0.034*	0.16	4.01	0.72	3.45
	5 yr.	431	0.12	-1.04	-0.07	0.411	-3.54	1.45	-2.81	0.72
MPA	3-4 yr.	804	0.11	0.78	0.13	0.060	0.03	1.59	0.23	1.33
	5 yr.	431	0.07	-0.39	-0.07	0.482	-1.47	0.70	-1.15	0.38
VPA	3-4 yr.	804	0.06	1.27	0.16	0.055	-0.03	2.56	0.35	2.18
	5 yr.	431	0.09	0.29	0.03	0.752	-1.48	2.05	-0.97	1.54
MVPA	3-4 yr.	804	0.06	2.07	0.16	0.029*	0.21	3.93	0.75	3.39
	5 yr.	431	0.09	-0.14	-0.01	0.914	-2.32	2.32	-1.87	1.60
**Full day**										
Total PA	3-4 yr.	757	0.05	-2.55	-0.02	0.850	-29.00	23.90	-21.31	16.21
	5 yr.	409	0.05	-33.07	-0.21	0.090	-71.37	5.24	-60.14	-5.99
SED	3-4 yr.	757	0.05	0.89	0.01	0.695	-3.54	5.31	-2.25	4.02
	5 yr.	409	0.02	4.55	0.07	0.125	-1.26	10.37	0.44	8.66
LPA	3-4 yr.	757	0.07	-0.77	-0.04	0.585	-3.52	1.99	-2.72	1.19
	5 yr.	409	0.02	-2.87	-0.13	0.112	-6.41	0.67	-5.37	-0.37
MPA	3-4 yr.	757	0.05	-0.33	-0.05	0.519	-1.35	0.68	-1.05	0.39
	5 yr.	409	0.02	-0.92	-0.12	0.196	-2.32	0.48	-1.91	0.07
VPA	3-4 yr.	757	0.02	-0.01	0.00	0.995	-1.55	1.54	-1.10	1.09
	5 yr.	409	0.04	-0.71	-0.07	0.526	-2.89	1.48	-2.25	0.84
MVPA	3-4 yr.	757	0.03	-0.26	-0.02	0.824	-2.59	2.07	-1.92	1.39
	5 yr.	409	0.03	-1.65	0.10	0.308	-4.84	1.53	-0.60	3.91

ICC=Intraclass correlation coefficient; ES=effect size. Analyses were adjusted for accelerometer wear time. Significant differences between groups are highlighted in bold. *Significant differences within groups at $p \leq 0.05$.

Additional file 7 A-B. Per-protocol analyses of A) FMS and FIT, and B) PA, based on attendance and delivery of written assignments.

A) FMS and FIT

			7-month follow-up			18-month follow-up		
	**N**	**ICC**	**Estimate (95% CI)**	**ES**	***p***	**Estimate (95% CI)**	**ES**	***p***
Locomotion	628	0.03	0.25 (-0.36–0.86)	0.06	0.425	0.90 (0.29–1.51)	**0.23**	**0.004**
Object control	631	0.03	0.10 (-0.48–0.68)	0.03	0.735	0.38 (-0.21–0.96)	0.13	0.205
Balance	629	0.06	-0.64 (-1.44–0.17)	-0.12	0.121	0.22 (-0.59–1.02)	0.04	0.594
Handgrip	629	0.02	-0.48 (-0.76–-0.20)	**-0.22**	**<0.001**	0.20 (-0.09–0.48)	0.09	0.174
Standing long jump	625	0.58	-1.68 (-4.82–1.46)	-0.07	0.294	-0.13 (-3.32–3.05)	-0.01	0.935
Motor fitness	628	0.02	-0.02 (-0.38–0.33)	-0.01	0.911	-0.14 (-0.49–0.22)	-0.05	0.454

ICC=Intraclass correlation coefficient; ES=effect size. FMS-outcomes were adjusted for FMS assessor. Statistical significance at p≤0.05 highlighted in bold.

B) PA

			7-month follow-up			18-month follow-up		
	**N**	**ICC**	**Estimate (95% CI)**	**ES**	***p***	**Estimate (95% CI)**	**ES**	***p***
**Preschool**								
Total PA	621	0.07	27.45 (-14.21–69.12)	0.14	0.196	-23.33 (-65.31–18.64)	-0.12	0.275
SED	621	0.12	-7.21 (-10.97–-3.46)	-0.29	**<0.001**	3.71 (-0.05–7.46)	0.15	0.053
LPA	621	0.17	3.96 (1.61–6.30)	0.26	**<0.001**	-3.26 (-5.61–-0.91)	-0.22	**0.007**
MPA	621	0.09	1.19 (0.22–2.17)	0.21	**0.016**	-1.04 (-2.02–-0.07)	-0.18	**0.036**
VPA	621	0.04	1.62 (0.06–3.17)	0.21	**0.042**	0.15 (-1.41–1.71)	0.02	0.848
MVPA	621	0.04	2.79 (0.59–4.99)	0.22	**0.013**	-0.93 (-3.13–1.27)	-0.07	0.409
**Full day**								
Total PA	585	0.03	-4.68 (-35.98–26.63)	-0.03	0.769	-11.78 (-42.53–18.97)	-42.53	0.452
SED	585	0.06	3.46 (-1.78–8.69)	0.05	0.195	4.97 (-0.20–10.14)	0.07	0.059
LPA	585	0.09	-2.61 (-5.93–0.72)	-0.12	0.125	-3.75 (-7.04–-0.47)	-0.17	**0.025**
MPA	585	0.04	-0.99 (-2.18–0.20)	-0.13	0.103	-1.28 (-2.46–-0.11)	-0.17	**0.032**
VPA	585	0.01	-0.21 (-2.05–1.62)	-0.02	0.819	-0.28 (-2.08–1.53)	-0.03	0.763
MVPA	585	0.02	-1.05 (-3.78–1.69)	-0.06	0.452	-1.44 (-4.13–1.26)	-0.09	0.295

ICC=Intraclass correlation coefficient; ES=effect size. analyses were adjusted for accelerometer wear time. Statistical significance at p≤0.05 highlighted in bold.

Additional file 8 A-B. Per-protocol analyses of A) FMS and FIT, and B) PA, based on preschools' evaluation of the extent to which ACTNOW became integrated in the everyday practice.

A) FMS and FIT

			7-month follow-up			18-month follow-up		
	**N**	**ICC**	**Estimate (95% CI)**	**ES**	***p***	**Estimate (95% CI)**	**ES**	***p***
Locomotion	643	0.04	0.48 (-0.11–1.08)	0.13	0.113	0.92 (0.32–1.53)	0.24	**0.003**
Object control	645	0.05	0.43 (-0.16–1.02)	0.14	0.150	0.10 (-0.49–0.69)	0.03	0.742
Balance	643	0.06	-0.64 (-1.45–0.18)	-0.12	0.128	-0.06 (-0.87–0.76)	-0.01	0.887
Handgrip	643	0.02	-0.33 (-0.60–-0.06)	-0.15	**0.017**	-0.17 (-0.44–0.10)	-0.08	0.222
Standing long jump	638	0.00	-0.97 (-4.06–2.13)	-0.04	0.540	-1.97 (-5.10–1.16)	-0.08	0.216
Motor fitness	642	0.05	0.16 (-0.17–0.48)	0.06	0.353	0.14 (-0.20–0.47)	0.05	0.424

ICC=Intraclass correlation coefficient; ES=effect size. FMS-outcomes were adjusted for FMS assessor. Statistical significance at p≤0.05 highlighted in bold.

B) PA

			7-month follow-up			18-month follow-up		
	**N**	**ICC**	**Estimate (95% CI)**	**ES**	***p***	**Estimate (95% CI)**	**ES**	***p***
**Preschool**								
Total PA	635	0.08	41.37 (0.17–82.57)	0.22	**0.049**	58.41 (17.10–99.72)	0.31	**0.006**
SED	635	0.08	-8.41 (-12.04–-4.78)	-0.35	**<0.001**	-5.97 (-9.58–-2.36)	-0.25	**0.001**
LPA	635	0.11	4.43 (2.16–6.70)	0.31	**<0.001**	1.59 (-0.67–3.85)	0.11	0.168
MPA	635	0.09	1.91 (0.95–2.87)	0.34	**<0.001**	1.60 (0.65–2.56)	0.29	**0.001**
VPA	635	0.05	1.82 (0.29–3.35)	0.24	**0.020**	2.54 (1.02–4.07)	0.33	**0.001**
MVPA	635	0.05	3.69 (1.52–5.86)	0.30	**<0.001**	4.08 (1.92–6.24)	0.33	**<0.001**
**Full day**								
Total PA	597	0.04	17.63 (-13.72–48.98)	0.13	0.270	56.44 (25.61–87.26)	0.41	**<0.001**
SED	597	0.04	-4.72 (-9.91–0.48)	-0.08	0.075	-8.37 (-13.48–-3.26)	-0.13	**0.001**
LPA	597	0.06	2.92 (-0.38–6.21)	0.14	0.083	3.27 (0.02–6.51)	0.16	**0.048**
MPA	597	0.04	0.96 (-0.24–2.15)	0.13	0.116	1.74 (0.57–2.91)	0.24	**0.004**
VPA	597	0.02	0.61 (-1.23–2.46)	0.07	0.514	3.13 (1.32–4.94)	0.33	**<0.001**
MVPA	597	0.02	1.62 (-1.12–4.37)	0.10	0.245	4.92 (2.22–7.61)	0.31	**<0.001**

ICC=Intraclass correlation coefficient; ES=effect size. analyses were adjusted for accelerometer wear time. Statistical significance at p≤0.05 highlighted in bold.

Additional file 9 A-B. Per-protocol analyses of A) FMS and FIT, and B) PA, based on researchers' experiences of overall commitment and management.

A) FMS and FIT

			7-month follow-up			18-month follow-up		
	**N**	**ICC**	**Estimate (95% CI)**	**ES**	***p***	**Estimate (95% CI)**	**ES**	***p***
Locomotion	663	0.04	0.38 (-0.21–0.96)	0.10	0.208	0.69 (0.11–1.28)	0.18	**0.021**
Object control	666	0.05	0.21 (-0.35–0.78)	0.07	0.462	0.33 (-0.24–0.90)	0.11	0.252
Balance	664	0.05	-0.44 (-1.21–0.33)	-0.08	0.261	0.60 (-0.17–1.36)	0.11	0.128
Handgrip	665	0.04	-0.50 (-0.77–-0.24)	-0.23	**<0.001**	0.32 (0.05–0.59)	0.15	**0.020**
Standing long jump	660	0.00	-1.28 (-4.28–1.71)	-0.05	0.401	-0.51 (-3.53–2.51)	-0.02	0.740
Motor fitness	663	0.03	0.10 (-0.24–0.43)	0.03	0.578	-0.05 (-0.39–0.28)	-0.02	0.753

ICC=Intraclass correlation coefficient; ES=effect size. FMS-outcomes were adjusted for FMS assessor. Statistical significance at p≤0.05 highlighted in bold.

B) PA

			7-month follow-up			18-month follow-up		
	**N**	**ICC**	**Estimate (95% CI)**	**ES**	***p***	**Estimate (95% CI)**	**ES**	***p***
**Preschool**								
Total PA	656	0.07	25.83 (-13.39–65.05)	0.13	0.196	-4.91 (-44.87–35.05)	-0.03	0.809
SED	656	0.12	-3.92 (-7.46–-0.37)	-0.17	**0.031**	3.35 (-0.25–6.96)	0.14	0.068
LPA	656	0.16	1.42 (-0.76–3.60)	0.10	0.202	-3.06 (-5.27–-0.84)	-0.21	**0.007**
MPA	656	0.11	0.69 (-0.23–1.61)	0.12	0.143	-0.75 (-1.68–0.19)	-0.13	0.119
VPA	656	0.04	1.59 (0.15–3.04)	0.20	**0.031**	0.21 (-1.26–1.68)	0.03	0.778
MVPA	656	0.05	2.32 (0.24–4.41)	0.18	**0.029**	-0.50 (-2.62–1.62)	-0.04	0.645
**Full day**								
Total PA	621	0.06	-10.88 (-40.80–19.04)	-0.08	0.475	13.34 (-16.57–43.24)	0.09	0.382
SED	621	0.07	3.58 (-1.44–8.60)	0.06	0.162	3.21 (-1.82–8.24)	0.05	0.210
LPA	621	0.08	-2.64 (-5.78–0.50)	-0.12	0.100	-3.23 (-6.38–-0.08)	**-0.15**	**0.045**
MPA	621	0.06	-0.95 (-2.10–0.21)	-0.13	0.107	-0.76 (-1.92–0.39)	-0.10	0.196
VPA	621	0.02	-0.17 (-1.92–1.58)	-0.02	0.848	0.62 (-1.13–2.36)	0.06	0.490
MVPA	621	0.04	-1.02 (-3.66–1.61)	-0.06	0.446	-0.08 (-2.72–2.57)	0.00	0.955

ICC=Intraclass correlation coefficient; ES=effect size. analyses were adjusted for accelerometer wear time. Statistical significance at p≤0.05 highlighted in bold.

Additional file 10. Regression coefficients of PA, FMS, and FIT change scores. Standardized regression coefficients of change scores (T3-T1) between PA, FMS, and FIT.

		Locomotor	Object control	Balance	Handgrip	Long jump	Motor fitness
**Preschool**	n	*529*	*555*	*531*	*570*	*483*	*533*
Total PA		0.13*	0.00	0.10*	0.05	0.10*	-0.087
SED		-0.09	0.04	0.00	-0.05	-0.13*	0.08
LPA		-0.04	-0.04	-0.08	0.00	0.09	-0.03
MPA		0.09	-0.05	-0.03	0.05	0.08	-0.09
VPA		0.16**	-0.01	0.09*	0.06	0.10*	-0.08
MVPA		0.16**	-0.03	0.06	0.07	0.10*	-0.10*
**Allday**	n	*438*	*461*	*444*	*479*	*396*	*438*
Total PA		0.08	0.03	0.06	-0.03	0.02	-0.03
SED		-0.11	0.00	-0.09	-0.02	-0.26*	0.16
LPA		-0.03	-0.02	0.01	0.01	0.11	-0.04
MPA		0.06	0.00	-0.01	0.02	0.09	-0.12*
VPA		0.11*	0.04	0.08	-0.01	0.05	-0.03
MVPA		0.10*	0.03	0.06	0.00	0.07	-0.07

Adjusted for accelerometer weartime. FMS outcomes adjusted for FMS assessor.
**Correlation is significant at ≤0.01. *Correlation is significant at ≤0.05.

Additional file 11

TIDieR Checklist

TIDieR
Template for Intervention Description and Replication

The TIDieR (Template for Intervention Description and Replication) Checklist*:

Information to include when describing an intervention and the location of the information

Item number	Item	Where located ** Primary paper (page or appendix number)	Other † (details)
	**BRIEF NAME**		
1.	Provide the name or a phrase that describes the intervention.	ACTNOW	
	**WHY**		
2.	Describe any rationale, theory, or goal of the elements essential to the intervention.	p.3-4	Protocol paper: https://www.ncbi.nlm.nih.gov/pmc/articles/PMC7350704/pdf/fpsyg-11-01382.pdf
	**WHAT**		
3.	Materials: Describe any physical or informational materials used in the intervention, including those provided to participants or used in intervention delivery or in training of intervention providers. Provide information on where the materials can be accessed (e.g. online appendix, URL).	p. 4-5 and additional file 1	https://activeinpreschool.com/
4.	Procedures: Describe each of the procedures, activities, and/or processes used in the intervention, including any enabling or support activities.	Additional file 1	Protocol paper: https://www.ncbi.nlm.nih.gov/pmc/articles/PMC7350704/pdf/fpsyg-11-01382.pdf
	**WHO PROVIDED**		
5.	For each category of intervention provider (e.g. psychologist, nursing assistant), describe their expertise, background and any specific training given.	p. 5-6 and additional file 1	Protocol paper: https://www.ncbi.nlm.nih.gov/pmc/articles/PMC7350704/pdf/fpsyg-11-01382.pdf
	**HOW**		
6.	Describe the modes of delivery (e.g. face-to-face or by some other mechanism, such as internet or telephone) of the intervention and whether it was provided individually or in a group.	p. 5-6 and additional file 1	Protocol paper: https://www.ncbi.nlm.nih.gov/pmc/articles/PMC7350704/pdf/fpsyg-11-01382.pdf
	**WHERE**		
7.	Describe the type(s) of location(s) where the intervention occurred, including any necessary infrastructure or relevant features.	p. 5-6 and additional file 1	

	**WHEN and HOW MUCH**		
**8.**	Describe the number of times the intervention was delivered and over what period of time including the number of sessions, their schedule, and their duration, intensity or dose.	p. 4-6	Intervention timeline, see protocol paper https://www.ncbi.nlm.nih.gov/pmc/articles/PMC7350704/pdf/fpsyg-11-01382.pdf
	**TAILORING**		
**9.**	If the intervention was planned to be personalised, titrated or adapted, then describe what, why, when, and how.	p. 5 and additional file 1	Protocol paper: https://www.ncbi.nlm.nih.gov/pmc/articles/PMC7350704/pdf/fpsyg-11-01382.pdf
	**MODIFICATIONS**		
**10.‡**	If the intervention was modified during the course of the study, describe the changes (what, why, when, and how).	p. 5 and additional file 1	
	**HOW WELL**		
**11.**	Planned: If intervention adherence or fidelity was assessed, describe how and by whom, and if any strategies were used to maintain or improve fidelity, describe them.	p. 7	Protocol paper: https://www.ncbi.nlm.nih.gov/pmc/articles/PMC7350704/pdf/fpsyg-11-01382.pdf
**12.‡**	Actual: If intervention adherence or fidelity was assessed, describe the extent to which the intervention was delivered as planned.	p. 10	

** **Authors** - use N/A if an item is not applicable for the intervention being described. **Reviewers** – use '?' if information about the element is not reported/not sufficiently reported.

† If the information is not provided in the primary paper, give details of where this information is available. This may include locations such as a published protocol or other published papers (provide citation details) or a website (provide the URL).

‡ If completing the TIDieR checklist for a protocol, these items are not relevant to the protocol and cannot be described until the study is complete.

* We strongly recommend using this checklist in conjunction with the TIDieR guide (see *BMJ* 2014;348:g1687) which contains an explanation and elaboration for each item.

* The focus of TIDieR is on reporting details of the intervention elements (and where relevant, comparison elements) of a study. Other elements and methodological features of studies are covered by other reporting statements and checklists and have not been duplicated as part of the TIDieR checklist. When a **randomised trial** is being reported, the TIDieR checklist should be used in conjunction with the CONSORT statement (see www.consort-statement.org) as an extension of **Item 5 of the CONSORT 2010 Statement.** When a **clinical trial protocol** is being reported, the TIDieR checklist should be used in conjunction with the SPIRIT statement as an extension of **Item 11 of the SPIRIT 2013 Statement** (see www.spirit-statement.org). For alternate study designs, TIDieR can be used in conjunction with the appropriate checklist for that study design (see www.equator-network.org).
